# Supplementary material for: A retrospective cohort study to observe the efficacy and safety of Endoscopic Submucosal Dissection (ESD) with adjuvant radiotherapy for T1a-MM/T1b-SM Esophageal Squamous Cell Carcinoma (ESCC)
Source: PLoS One. 2024 Feb 22;19(2):e0298792. doi: 10.1371/journal.pone.0298792 (PMC10883569; doi:10.1371/journal.pone.0298792)
Supplement: Attachment — Submitted filename: Response to Reviewers (v2.3 Jan-19-2024).pdf [file pone.0298792.s002.pdf]

Reviewers' comments:

Reviewer's Responses to Questions

Reviewer #1: Though it is a retrospective study, still some questions are there to find out.
1. It will remain a question to authors why radiotherapy is used in case of T1a-MM without LVSI.

Thank you for the feedback.

In the background, it has been mentioned that for patients with T1a-MM esophageal squamous cell carcinoma without vascular invasion after endoscopic resection, the metastasis rate in the follow-up observation group is 5.6%. However, considering that additional surgical resection may result in a decreased quality of life and potential treatment-related mortality, as well as the delayed adverse events and treatment-related mortality associated with additional chemoradiotherapy, the guidance committee did not reach a conclusion on whether to recommend additional treatment.

2. What is craniocaudal margin used in radiotherapy portal.

The margin is described in the radiotherapy section: "A titanium clip was endoscopically placed in the tumor bed (post-ESD scar) before the localization CT to indicate the upper and lower borders. The GTVtb defined as the upper and lower esophageal border was determined by these titanium clips, and the PGTVtb was the 1 cm outward expansion of GTVtb."

3. Grades of Radiotherapy toxicity (Grade 1,2,3) data will be interesting to know.

Thank you for the feedback, we revised the term according to the criteria listed in the Common Terminology Criteria for Adverse Events, version 4.0.

Reviewer #2:

1. Its better to replace word "additional" with "Adjuvant" in Title and elsewhere

Thank you for your feedback. We have made revisions accordingly.

2. Abstract- line 25, replace received with underwent.

Thank you for your feedback. We have made revisions accordingly.

3. Introduction

a. Rewrite 45-48 line as your data do not match with reference

Thank you for your observation. The data used in lines 45-48 is derived from reference 4, and we have modified it and changed the citation. We'll be happy to revisit and make adjustments as needed. If you have any specific concerns or suggestions, please feel free to share.

(4) Ishihara R, Arima M, Iizuka T, Oyama T, Katada C, Kato M, et al. Endoscopic submucosal dissection/endoscopic mucosal resection guidelines for esophageal cancer. Dig Endosc. 2020 May;32(4):452–93.

b. Reference 10, may not exactly show LN mets incidence cause its done as salvage so may not be appropriate reference here

Thank you for the feedback. Upon review, we agree that this reference does not suit this context and, as a result, it has been removed.

c. Rewrite sentence 61-63

Thank you for your feedback. We have made revisions accordingly to have a clearer view of the background and the guidelines.

d. In Radiotherapy section, in line 128, was Left Anterior descending artery contour as OAR? It is considered most important OAR in heart.

Thank you for your feedback. We consider Left Anterior descending artery as OAR, and have made revisions accordingly.

e. Dose of Radiotherapy have differed significantly (41.4 Gy vs 50.4Gy). how was dose decided?

The radiotherapy doses have been determined based on historical references, and we have incorporated this information in the methods section.

f. In follow up section, How frequently was CT scan done?

Thank you for the feedback, CT was done every 6 months during the first year after gastroscopy, and then annually afterward.

g. In result section, complication should be reported based on grade rather than using word like mild.

Thank you for the feedback, we revised the term according to the criteria listed in the Common Terminology Criteria for Adverse Events, version 4.0.

h. In discussion section, since it is retrospective study of small sample rather than comparing RT vs Non RT group, it is better for author to conclude that adjuvant RT is safe and effective with good results

Thank you for the feedback, we have made revisions accordingly.
