# Supplementary material for: A retrospective cohort study to observe the efficacy and safety of Endoscopic Submucosal Dissection (ESD) with adjuvant radiotherapy for T1a-MM/T1b-SM Esophageal Squamous Cell Carcinoma (ESCC)
Source: PLoS One. 2024 Feb 22;19(2):e0298792. doi: 10.1371/journal.pone.0298792 (PMC10883569; doi:10.1371/journal.pone.0298792)
Supplement: Attachment — Submitted filename: Response to Reviewers.docx [file pone.0298792.s001.docx]

Dear Ms.Gomes,

I am writing to offer my sincerest thanks for your response and sincere apologies for not providing a proper response about the data source to the reviewer's question in our recent communication. It was not our intention to neglect or overlook the question, and I take full responsibility for this lapse in our communication.

We value your input and feedback, and it is essential for us to address all inquiries promptly and thoroughly. I want to assure you that we are committed to rectifying this situation immediately. We have reviewed the question in detail and have now prepared a comprehensive response to address your concerns.

Our data was obtained from the Ningbo Medical Center Lihuili Hospital and the data collection period was from January 1, 2016 through December 31, 2020. Due to the COVID-19 pandemic from 2021 to 2022, the medical center and medical utilization were used priority for patients with COVID-19 positive, so we didn't apply for the data during the COVID-19 pandemic. Furthermore, the most important is that this study was approved by the Ethics Committee of Biomedical Research Involving Humans of Ningbo Medical Center Lihuili Hospital on March 16, 2023 (IRB number: KY2023SL054-01). And we received the data from a hospital-based release on March 30, 2023 after the IRB approval without any human rights concerns.

All raw data cannot be shared publicly because of personal data protection and privacy of the patients. Data are available from the Ningbo Medical Center Lihuili Hospital Ethics Committee for researchers who meet the apply criteria for access to confidential data.

Once again, I apologize for any inconvenience this may have caused, and I want to thank you for your patience and understanding in this matter. We appreciate the time and effort you dedicated to reviewing our work. Your insights have been incredibly beneficial in improving the quality of our research. We truly value your expertise in the field. We kindly request your permission to resubmit the revised manuscript to PLOS ONE. We are confident that the improvements made will contribute to the scientific community and enhance the quality of the journal.

Your approval to resubmit to PLOS ONE would be greatly appreciated. We are committed to ensuring that our work meets the high standards set by the journal.

Please feel free to reach out if you have any further questions or if there is anything else we can do to assist you. We greatly appreciate your feedback.

Best Regards,

Xuesong Zhang
